# Hospital Saturday

**Published:** 1887-06-11

**Authors:** 


### HOSPITAL SATURDAY.

There is positive pleasure in witnessing vast numbers of men of all ranks and conditions concentrating their united energies on a great and worthy object. The mere fact of their agreement is wholesome and pleasant. There is such an evident disposition on the part of modern peoples to "bite and devour one another," that the peace-loving citizen looks upon any uniting institution with fervent gratitude.

There was a time when the Hospital Saturday movement was a bone of contention, a lighted match held near a powder-magazine. To some people the mention of it was as a red rag to a bull. That is over; and the Fund has slowly but surely won its way to public confidence and favour. The strife, however, like all conflicts, has left some traces behind. The managers of the Fund, having been very freely handled in the way of criticism, are inclined to be a little sensitive, and perhaps to proceed with cautious timidity in a direction where a bold advance would be more likely to gain the desired end. Timidity is very pleasant in bashful young ladies, and caution is desirable where experience is small; but in tried and accredited public movements, about the value of which there can hardly be two opinions, a confident and courageous line of conduct is the assured way of success.

The Saturday Fund has no reason to blush for its record. True, the results it has so far produced are by no means magnificent. But in estimating the success of agricultural operations it is necessary not only to consider the value of the crop, but also the nature of the soil, the means of cultivation, the capital in hand, and a variety of circumstances which go to make up the history of the subject. The Saturday Fund has had the poorest and most uncultivated philanthropic soil to deal with which could well be imagined. Nobody can think the London workshop favourable ground for philanthropy, or even for rational self-help. The income of the average workman is too small, and his necessary expenses too large, for him to have much to spare, either for charity or for a rainy day provision. His habits, which are the habits of generations of his ancestors; and the temptations to low pleasures which abound on all hands, drain his resources still further, and deteriorate his character at the same time. Yet this is the kind of soil the Saturday Fund has had to put its plough into. And if, as we have said, the crop as yet is not large, it is essential to remember that only after years of careful culture can such soil hope to become fruitful.

Fruitful, however, it is rapidly becoming, thanks to the pluck and perseverance of those who have had the Fund in hand. The fact that the first street collection, thirteen years ago, amounted to no more than two hundred and fifty-eight pounds, whilst the same collection last year exceeded four thousand, is more eloquent than any laboured argument in proof of progress. Of course, the street collection is but a part of the movement, and not the most important part. The workshop contributions are a far more significant feature, and are rapidly increasing. These collections are taken weekly or monthly, for a period of six months annually, viz., from April to September. The steady and systematic working of this part of the Fund may yet be productive of vast and unexpected results. There are now about three thousand business firms at whose establishments these collections are systematically taken. In this year of Jubilee an effort is to be made to raise the number to twenty thousand. We counsel the managers to devote all possible intelligence and energy to this branch of their work. If wisely developed, it should become a perennial mine of gold for the support of the medical charities.

The street collection is to be conducted this year on a scale of unparalleled magnitude. From Croydon in the south to Wood Green in the north, and from Brentford in the west to Forest Gate in the east, the lady pleaders will line the public roads and solicit the help of the charitable. Strange and gratifying spectacle to see at least fifty miles of streets in a straight line, as well as an incalculable mileage in the metropolis itself, occupied by those patient sisters of humanity who seek the well-being of the poor and sick!

The London cabmen this year are determined to be well to the front. Each Jehu is to have his whip adorned with a gaily-coloured pennon, and the window of every cab will be eloquent with a suitable poster. Selected and responsible cabbies will be provided with collecting-boxes, and every fare will be expected to give at least a silver *douceur* for the box, in addition to the driver's due and proper reward.

Special collections are to be taken at the works of all the great railway companies, at the police-stations, and at the post-office. The various vestries, working-men's clubs, friendly and temperance societies, are uniting as one man to give a grand impetus to the movement.

It is astonishing to think of the energy and generalship which have been put forth in order to move all these vast and various bodies simultaneously in one direction. As we said before, the results seem comparatively insignificant for such wonderful preparations. But this is only the seed-time of the movement: the full harvest will come by-and-by.

By way of striking the public imagination once for all, a monster demonstration is to be held in Victoria Park. If the people should be favoured with fine weather this will be a genuine and splendid carnival of charity. Nothing could be better or more worthy of this year of Jubilee in honour of which the demonstration is to be held. The principal temperance and benefit societies will march past, robed in the costumes of the various Kings of England from the earliest dawn of history. Never before was such a spectacle seen or contemplated on a similar occasion. Most heartily we wish the demonstrators the fullest measures of success which their cause merits and their hearts can desire.

At no previous period has the Saturday Fund put forth such vast and splendidly organised efforts; and we hope the results will be tenfold more than their most sanguine anticipations. Should this be so, a stimulus will be given to the Sunday Fund, and to the Hospital movement generally, which may make the year of Jubilee the most memorable in their history. What other object can be so great, so noble, and so worthy of all men's sympathy as the care and restoration to health of those who, but for the hospital and kindred institutions, would in times of sickness have neither help nor hope?

